# Mental Health Care Provider Experiences of Remote Measurement-Based Care Rollout in an Urban Safety-Net Psychiatry Department: Three-Site Mixed Methods Hypothesis-Generating Implementation Study

**DOI:** 10.2196/71570

**Published:** 2025-09-05

**Authors:** Ana M Progovac, Carl Fulwiler, Margaret Lanca, Daisy Wang, Kate Zona, Norah Mulvaney-Day, Rajendra Aldis, H Stephen Leff, Philip Wang, Lisa C Rosenfeld

**Affiliations:** 1 Department of Psychiatry Cambridge Health Alliance Cambridge, MA United States; 2 Department of Psychiatry Harvard Medical School Boston, MA United States; 3 Institute for Excellence in Health Equity NYU Langone Health New York, NY United States

**Keywords:** measurement-based care, Collect-Share-Act model, therapeutic alliance, implementation science, safety net, mental health services

## Abstract

**Background:**

Measurement-based care (MBC), including remote MBC, is increasingly being considered or implemented for mental health treatment and outcomes monitoring in routine clinical care. However, little is known about the health equity implications in real-world practice or the impact on patient-provider relationships in lower-resource systems that offer mental health treatment for diverse patients.

**Objective:**

This hypothesis-generating study examined the drivers of MBC implementation outcomes, the implications for health equity, and the impact of MBC on therapeutic alliance (TA). The study was conducted 1 year after the implementation of remote MBC at 3 outpatient adult clinics in a diverse, safety-net health system.

**Methods:**

This explanatory sequential mixed methods study used quantitative surveys and qualitative focus groups with mental health care providers. Repeated surveys were first used to understand mental health care provider experiences over a 6-month period, at least 1 year after MBC implementation. Surveys were analyzed to refine focus group prompts. Six mental health providers participated in repeated surveys over 6 months, after which the same 6 providers and 1 additional mental health provider took part in focus groups.

**Results:**

Surveys revealed stable acceptability and utility ratings, concerns that MBC was not equally benefiting patients, little endorsement that MBC improved TA, and slightly decreasing feasibility scores. In focus groups, mental health care providers shared concerns about the acceptability, appropriateness, feasibility, and equity of processes for collecting MBC data. These providers had less first-hand experience with sharing and acting upon the data but still voiced concerns about the processes for doing so. TA both impacted and was impacted by MBC in positive and negative ways. The potential drivers of the findings are discussed using qualitative data.

**Conclusions:**

More than 1 year after the implementation of remote MBC for mental health, mental health care providers had enduring concerns about its implications for health equity as well as its bidirectional relationship with TA. These findings suggest that further study is needed to identify system-level strategies to mitigate potential negative effects of real-world MBC implementations on health equity, particularly in low-resource settings with diverse populations.

## Introduction

### Background

Measurement-based care (MBC) is an evidence-based approach that uses repeated collection of standardized, quantitative data to track progress on care goals and inform treatment planning [1,2]. In the mental health field, many types of measures can be used, including symptom screeners (eg, Patient Health Questionnaire-9), quality of life measures (eg, World Health Organization Quality of Life Brief Version), and functioning scales (eg, Practical and Social Functioning Scale). Regardless of the measure used, practicing MBC implies not only capturing trends in health data but also using these data in session to understand how the patient is doing and make shared decisions about next steps in care.

MBC, which has been used across a variety of mental health settings, has been linked to benefits such as improved health outcomes [3-6] and proposed as a means to reduce health disparities [7-9]. MBC may lead to improved outcomes compared to treatment as usual because it allows for more standardized communication between patients and health care providers [4]. Furthermore, MBC has the potential to enhance therapeutic alliance (TA; also referred to as “working alliance”), which is often considered a key mechanism for treatment success [10,11]. TA refers to the shared emotional bond, understanding of treatment goals, and conceptualization of the tasks needed to address these goals shared by patient and mental health care provider [12]. Potential mechanisms for MBC’s positive impact on health disparities include reducing certain forms of bias in clinical judgment as well as improving health systems’ abilities to identify differential needs and intervention effectiveness among patient subpopulations [4,13].

Despite its promise, MBC adoption in mental health remains low [4,14-18]. A survey of >800 psychologists published in 2007 who were members of the American Psychological Association found that 62.9% used no validated outcome measures in their work with patients [16]. An evaluation of >300 American psychiatrists published in 2008 revealed that a majority never or rarely used standardized scales, while just 6.5% used scales almost all the time [18]. As recently as 2018, a study of survey responses from >500 mental health counselors, marriage and family therapists, and social workers who were members of their respective professional societies found that the needle had not moved, with 61.5% never using validated outcome measures [17].

The Collect-Share-Act (CSA) model was developed as a framework to support mental health care provider adoption of MBC [19]. CSA breaks MBC into 3 distinct stages, each of which must be followed in sequence before progression to the next. In the *Collect* stage, mental health care providers are tasked with introducing MBC to patients and explaining the rationale for its use, then measuring the agreed-upon outcome repeatedly. In the *Share* stage, mental health care providers are asked to share and reflect on the measurement results with patients. In the *Act* stage, mental health care providers are directed to assess the patient’s longitudinal trajectory and collaboratively determine next steps.

Most research on the use of MBC in mental health care has focused on relatively homogeneous patient populations with lower-acuity conditions, typically receiving care in small, stand-alone practices [20]. Furthermore, most work linking MBC with reductions in health disparities remains theoretical [21]; and national experts agree that there is no consensus about whether MBC, as currently designed and implemented, can assist with targeting and reducing mental health care disparities [22].

The paucity of data from higher-need, more diverse health systems raises the question of whether existing MBC literature applies to safety-net settings. The data that do exist suggest that MBC uptake is lower among older versus younger, racial and ethnic minority versus White, male versus female, severely versus nonseverely ill, and publicly versus privately insured individuals [3,23,24]. As patients with different demographic or diagnostic profiles may encounter group-specific challenges at any of the CSA stages of MBC, understanding whether and where these difficulties arise is essential for helping mental health care providers and mental health systems ensure access to the evidence-based practice of MBC for all patients.

Similarly, the relationship between MBC and TA in diverse real-world settings remains speculative in nature [22]. Evidence from randomized controlled trials and meta-analyses suggests that a strong TA between patients and their psychiatric providers improves mental health outcomes and that using MBC enhances patient-provider communication and hence shared decision-making [25-28]. However, to date, little is known about whether MBC enhances TA for patients from minoritized groups or whether strong TA can be leveraged to increase patient engagement with MBC. Demonstrating that MBC has a positive impact on TA among mental health care providers working with diverse patient populations could enhance buy-in among safety-net clinicians. Conversely, exploring the challenges that MBC poses to TA in these settings may allow mental health clinicians to anticipate and more effectively address barriers as well as remain committed to the evidence-based approach to care.

### Objectives

In this explanatory sequential mixed methods study, we elicited feedback 1 year after the centralized implementation of an MBC tool called the Computerized Adaptive Test for Mental Health (CAT-MH). The study included 7 mental health care providers working with patients in an urban, ambulatory psychiatry department. Given the relative dearth of research exploring the on-the-ground realities of implementing MBC in safety-net health systems, the findings from this rich, small-scale study were intended to be hypothesis generating. Our goal was to better understand key phenomena that may drive disparate MBC uptake and varying real-world impact of MBC in safety-net settings. Specifically, we explored whether mental health care providers found using MBC in these settings acceptable (ie, whether they liked using it with patients), appropriate (ie, whether they felt that it was relevant and compatible with their practice and patient population), and feasible (ie, whether they found it easy to implement in their practice) [29]. On the basis of unanswered questions in existing literature and our prior studies, we also sought to clarify how these implementation phenomena are perceived within each CSA stage and how MBC impacts health equity and TA.

## Methods

### Overview

This explanatory sequential mixed methods hypothesis-generating study used 3 repeated surveys over 6 months to understand provider experiences with MBC implementation and inform the focus group guide for the subsequent discussions. An overview of the study design is presented in Figure S1 in Multimedia Appendix 1 [19,30]. The results are jointly interpreted in the Discussion section, where potential avenues for further research are also presented.

### Participants and Setting

This study was conducted in a diverse US urban safety-net system and training institution oriented toward team-based and psychosocial approaches to care. Patients receiving mental health services in this system were predominantly publicly insured, and many experienced chronic conditions that led to ongoing engagement in psychiatric care over the course of years.

All psychologists, social workers, psychiatrists, and psychiatric nurse practitioners who provided direct patient care at 3 adult-serving clinics were eligible for participation in the study. These included a general outpatient clinic, an addiction-focused clinic, and a transitional clinic that served individuals moving from inpatient to outpatient psychiatric care. Additional details on the demographic and diagnostic groups that each of these clinics served are provided in a prior publication [31].

These clinics were selected for inclusion in our study because they had been the sites of a centralized effort to roll out MBC in outpatient psychiatry in the year prior (2021). The providers at these clinics were expected to use session time to review MBC data with patients to assess their clinical progress and make shared decisions regarding next steps in care. The providers who were eligible for this study had at least 1 full year of experience with MBC after the implementation start date.

The MBC rollout involved the use of the CAT-MH, which has been validated against structured clinical interviews and shown to have high reliability and validity [32-34]. The CAT-MH draws from an item bank of nearly 1500 questions and uses multidimensional item response theory to assess 8 symptom domains [35]: depression, anxiety, mania and hypomania, psychosis, substance use disorder risk, posttraumatic stress disorder, attentional disorders, and a pilot quality-of-life module. Overall, the CAT-MH is reported to take about 2 minutes per domain to complete. The CAT-MH was delivered asynchronously through an app- or web-based electronic patient portal before an outpatient visit, with a minimum interval of 8 weeks between assessments. Module scores, including a severity indicator, were available on the electronic patient portal and electronic medical record. Individual item responses were not available for review once the survey was complete. Providers could graph the CAT-MH results using the electronic health record to see changes over time, although patients could not independently view their trend lines. Patient completion rates for MBC assessments in the first 6 months of implementation were 38% in the general outpatient clinic, 28% in the addiction-focused clinic, and 17% in the transitional clinic [31]. More detailed information on MBC completion trends is available elsewhere [31].

A key motivation for our study, which began in 2022 (a full year after the MBC rollout), was to understand how providers were experiencing MBC. Exposure to MBC came through 1-on-1 clinical work with patients, case discussions in weekly team meetings, and sporadic department-wide presentations (eg, grand rounds), some of which continued into early 2022. As implementing MBC is a complex process, we anticipated that it would take time for providers and patients to adjust to new workflows and that providers would have a better assessment of how these processes were impacting their work with patients, especially with regard to health equity, only after sufficient time had elapsed.

### Ethical Considerations

This study received institutional review board approval from Cambridge Health Alliance (approval number CHA-IRB-20-21-6). Consistent with institutional policies at Cambridge Health Alliance, providers were not compensated financially for their participation in the study; however, they were permitted to block time in their work schedule to participate in the study. All participants gave written informed consent via DocuSign and were informed that study data would be shared anonymously.

### Research Team

The research team was led by a health services researcher with PhD training (AMP) and a psychiatrist with an MD degree (LCR). It also included 3 other psychiatrists with MD degrees (CF, RA, and PW), 3 psychologists with PhD degrees (ML, KZ, and HSL), another PhD-trained health services researcher (NM-D), and an undergraduate research assistant (DW). Study leads AMP and LCR were trained in quantitative and mixed methods research in health services and implementation science. The study leads were women; the rest of the research team included 4 men and 4 women. At the time of the study, all members of the research team were employed by the safety-net health system as clinicians, researchers, clinical leaders, or part-time research assistant interns. Although the researchers had passing professional contact with study participants, only the study leads (AMP and LCR) and the research assistant (DW)—who had no supervisory roles related to participants—had direct contact with participants, knew their identities, or handled their identifiable data throughout the study duration. The study team had previously announced ongoing MBC evaluation efforts to all providers at the eligible sites, and these goals were explained again during recruitment.

### Recruitment

All mental health care providers at the 3 sites were invited to participate via emails sent to each team. The study leads (AMP and LCR) also attended team meetings to introduce the study. The goals of the study, along with its voluntary nature, were reiterated in individual consent forms and in optional additional individual informed consent meetings.

### Measures and Focus Groups

#### Repeated Surveys

Providers completed 3 identical 10- to 15-minute electronic surveys (via Google Forms) in May, July, and September 2022. The surveys included closed- and open-ended questions about MBC acceptability, adoption, feasibility, utility, equity, sustainability, and impact on TA (refer to the Results section for the survey items and question sources). Structured items were informed by the conceptual model of implementation research developed by Proctor et al [29], the implementation outcome measures formulated by Weiner et al [36], and the Working Alliance Inventory–Therapist version [37,38].

#### Qualitative Focus Groups

Three 1-hour web-based focus groups, each including 2 to 3 providers, were conducted between October and November 2022. They were led by the study leads (AMP and LCR) and designed to elicit narratives around key survey findings related to acceptability, appropriateness, feasibility, and equity within the CSA stages [19] as well as the impact of MBC on TA. The focus groups were recorded and transcribed verbatim (audio only). Transcripts were not returned to participants for correction but were quality checked by the first author (AMP). The study leads (AMP and LCR) compiled field notes after each session. The focus group guide was pilot-tested within the study team. No focus groups were repeated. Data saturation and informational richness [39] were prioritized via the longitudinal, explanatory mixed methods study design with key informant providers.

### Mixed Methods Analysis

#### Quantitative Data Analysis to Inform Focus Groups

Survey data were collected via Google Forms. Although means and SDs were calculated using Google Sheets, quantitative data were not used for hypothesis testing. Instead, the researchers qualitatively assessed the distribution of individual responses, with no formal assessment of statistical significance. Descriptive assessment of survey responses across all 3 rounds informed the focus group guide. Examples of how individual items were qualitatively assessed are shown in Figure S2 in Multimedia Appendix 1, and how patterns of responses were used to inform the focus group guide are shown in Table S1 in Multimedia Appendix 1 (both of which are described in detail in the Data Integration subsection).

#### Qualitative Focus Group Data Analysis

Qualitative transcripts and memos were reviewed to generate preliminary thematic summaries and inform code tree development. Subsequently, the study leads (AMP and LCR) and the research assistant (DW) developed a code tree and code book with input from key study team members. The final code tree consisted of both deductive (a priori) codes based on the focus group guide and inductive codes, highlighting important emerging concepts (Table S2 in Multimedia Appendix 1).

#### Focus Group Coding, Including Valence Coding

Valence coding helps summarize and compare the influence of constructs on implementation outcomes [40]. Two primary coders (first author AMP and research assistant DW) coded focus group excerpts by applying up to 2 codes each for up to 4 structured levels in a Google Sheet (refer to Table S2 in Multimedia Appendix 1 for additional definitions). All excerpts were assigned a level 1 code (CSA or TA). For excerpts coded as a CSA stage at level 1, subsequent levels were as follows: level 2—acceptability, appropriateness, feasibility, or equity; and level 3—a valence code (−1, 0, +1, or N/A) to indicate how data from each excerpt impacted the implementation outcomes. For excerpts coded as TA at level 1, subsequent levels were as follows: level 2—MBC’s impact on TA or TA’s impact on MBC; and level 3—a valence code (−1, 0, +1, or N/A) to indicate the direction of impact (positive or negative).

Level 4 codes captured CSA subdomains, new inductive codes, and detail regarding the nature of MBC’s relationship to TA. Codes were only applied where relevant (ie, could be left blank). To finalize the code tree and definitions, sample excerpts were also coded and reviewed by a third coder (senior author LCR), and additional input was obtained from the qualitative team during regular research meetings. For final coding, the 2 primary coders independently coded all excerpts; identified areas of coding incongruence; and resolved discrepancies through discussion, where possible. The third coder (senior author LCR) helped resolve remaining coding discrepancies via consensus. Final consolidated coding sheets contained up to 2 codes per excerpt.

### Data Integration

The goal of this explanatory sequential mixed methods hypothesis-generating study was to generate a richer understanding (rather than to test hypotheses) of MBC implementation challenges at each CSA stage and how these challenges impact health equity and TA. Data integration occurred at 2 time points. First, provider surveys informed the focus group guide, including the selection of specific CSA substeps around which focus group prompts would be generated, the decision to include questions about the health equity implications at each CSA stage, and the decision to include targeted TA prompts to understand the direction and nature of the relationship between MBC and TA. Second, the qualitative data were used to explain and expand upon the observed quantitative findings.

### Strategies to Ensure Data Trustworthiness

Our study incorporated 8 out of 10 recommended strategies for ensuring trustworthiness in qualitative research [41]. We used all 3 recommended strategies to ensure credibility, both recommended strategies to enhance transferability, and both recommended strategies to enhance dependability. While we only formally applied 1 of the 3 recommended strategies to ensure confirmability (ie, peer debriefing), our focus groups drew inspiration from group member checking, in that we shared brief summaries of group-level findings from the quantitative data with focus group participants. This allowed the opportunity for providers to react to these group-level findings and offer contrasting perspectives during the focus groups.

## Results

### Participant Characteristics

Of the 26 eligible mental health care providers, 7 (27%) chose to participate in the study. Of these 7 participants, 1 (14%) chose to participate only in the focus group. Participants included psychologists, social workers, psychiatrists, and psychiatric nurse practitioners at 3 outpatient adult clinics. They were primarily female, White, and aged between 25 and 54 years; all provided care in English (Table 1). Participants had at least 1.5 years of experience in their roles at the organization before enrolling in this study, which meant that they were all involved in the MBC rollout for the full year before our study began.

**Table 1:** Participant characteristics (n=6^a^).

Characteristics			Participants, n (%)
**Gender**			
	Men	1 (17)	
	Women	5 (83)	
	Nonbinary	0 (0)	
**Primary languages spoken at home**			
	English	6 (100)	
**Primary languages used in patient care**			
	English	5 (83)	
	English and Spanish	1 (17)	
**Race**			
	White	6 (100)	
**Ethnicity**			
	Non-Hispanic	6 (100)	
**Duration of employment at organization (years)**			
	<5	3 (50)	
	≥5	3 (50)	
**Specialization**			
	Clinical psychology or psychiatry^b^	3 (50)	
	Social work	3 (50)	

^a^Of the 7 participants, 1 (14%) did not wish to provide demographic information.

^b^Psychiatry includes physicians and nurse practitioners.

### Quantitative Surveys

All survey questions, question sources, and results from all 3 rounds of the quantitative surveys are presented in Table 2. All responses were on a Likert scale from 1 (completely disagree) to 5 (completely agree), unless otherwise noted.

Acceptability scores largely remained stable across the 3 rounds (round 1: mean 3.0, SD 0.7; round 2: mean 3.5, SD 0.6; and round 3: mean 3.3, SD 0.5). In examining adoption, only 1 (17%) of the 6 mental health care providers reported “never” reviewing CAT-MH results with patients over time, and providers generally reviewed CAT-MH results with patients only when new data were available (round 1: 4/5, 80%; round 2: 3/6, 50%; and round 3: 5/6, 83%). Mental health care providers were mostly evenly split between not reviewing CAT-MH results at all before visits (round 1: 2/5, 40%; round 2: 3/6, 50%; and round 3: 3/6, 50%) and spending 1 to 5 minutes reviewing these results (round 1: 3/5, 60%; round 2: 3/6, 50%; and round 3: 3/6, 50%). By round 3, most of the mental health care providers (4/6, 67%) reported spending 1 to 5 minutes during the patient visit reviewing CAT-MH results, whereas in round 1 there was a more even split across categories.

**Table 2:** Quantitative provider feedback on the implementation of the Computerized Adaptive Test for Mental Health (CAT-MH) implementation based on 3 surveys administered every 2 months between May and September 2022^a^.

Domains and survey questions				Round 1 (n=5)		Round 2 (n=6)		Round 3 (n=6)
**Acceptability, mean (SD)**								
	“I like reviewing CAT-MH data with patients”		3.0 (0.7)		3.5 (0.6)		3.3 (0.5)	
**Adoption, n (%)**								
	“**I review CAT-MH results with patients”**							
		“Never”	0 (0)		1 (17)		0 (0)	
		“When there are new results”	4 (80)		3 (50)		5 (83)	
		“Only when the patient brings it up”	1 (20)		2 (33)		1 (17)	
		“At regular intervals”	0 (0)		0 (0)		0 (0)	
	“**I spend approximately __ minutes reviewing CAT-MH results for a patient before the visit”**							
		0	2 (40)		3 (50)		3 (50)	
		1-5	3 (60)		3 (50)		3 (50)	
		5-10	0 (0)		0 (0)		0 (0)	
		>10	0 (0)		0 (0)		0 (0)	
	“**I spend approximately __ minutes reviewing CAT-MH results for a patient during the visit”**							
		0	1 (20)		1 (17)		1 (17)	
		1-5	2 (40)		5 (83)		4 (67)	
		5-10	2 (40)		0 (0)		1 (17)	
		>10	0 (0)		0 (0)		0 (0)	
**Utility, mean (SD)**								
	“I trust the data generated by CAT-MH”		2.8 (0.8)		3.0 (0.9)		2.8 (0.4)	
	“CAT-MH data increases my confidence in a patient’s diagnosis”		2.8 (0.8)		3.8 (0.4)		3.5 (0.8)	
	“CAT-MH data helps me and my patients set priorities for our visits”		2.8 (1.1)		3.5 (0.5)		3.3 (1.2)	
	“CAT-MH data impacts my treatment recommendations”		2.6 (1.3)		3.5 (0.5)		3.2 (1.0)	
	“Reviewing CAT-MH data during visits helps me and my patients collaborate on treatment decisions”		3.0 (1.2)		3.2 (0.8)		3.3 (0.8)	
	“CAT-MH data helps me assess whether a patient is responding to treatment”		2.8 (1.3)		3.3 (0.8)		2.7 (1.2)	
**Equity,** **mean (SD)**								
	“I believe that CAT-MH is benefitting all of my patients equally”		1.4 (0.5)		1.3 (0.5)		1.5 (0.5)	
**Therapeutic alliance,** **mean (SD)**								
	“Using CAT-MH has helped me feel like I better understand my patients”		2.6 (0.9)		3.0 (1.3)		3.0 (0.6)	
	“Using CAT-MH has helped me and my patients develop a common understanding of our treatment goals”		2.4 (1.1)		2.8 (1.2)		2.8 (1.0)	
	“CAT-MH has helped me and my patients build mutual trust”		1.6 (0.5)		2.3 (0.8)		2.3 (0.8)	
**Feasibility,** **mean (SD)**								
	“Discussing CAT-MH results with patients is doable in the time we have for visits”		3.6 (0.5)		3.5 (0.5)		3.3 (0.8)	
**Sustainability,** **mean (SD)**								
	“I believe that, over time, the benefits of using CAT-MH will outweigh the challenges of learning to use a new tool”		3.2 (0.4)		3.7 (0.5)		3.3 (0.5)	
	“I expect that, over time, incorporating CAT-MH into my day to day work will become: 1 (a lot harder) to 5 (a lot easier)”		3.4 (0.5)		3.8 (0.4)		3.7 (0.5)	

^a^Unless otherwise noted, all Likert scale responses ranged from 1 (completely disagree) to 5 (completely agree), with a score of 3 (neither agree nor disagree) representing a neutral response. These data were assessed qualitatively to inform and refine the qualitative focus group guide and not for hypothesis testing; therefore, no formal statistical analysis is included here. Questions related to acceptability and feasibility were adapted from the Acceptability of Intervention and Feasibility of Intervention measures [36]. Questions related to therapeutic alliance were adapted from the Working Alliance Inventory–Therapist version: “I feel like I really understand [patient name],” “[Patient name] and I have a common perception of his/her goals,” “[Patient name] and I have built a mutual trust.” The Working Alliance Inventory is described in Horvath and Greenberg [37].

The utility questions revealed that mental health care provider trust in CAT-MH data was largely neutral over time (round 1: mean 2.8, SD 0.8; round 2: mean 3.0, SD 0.9; and round 3: mean 2.8, SD 0.4). By survey rounds 2 and 3, these providers generally agreed that CAT-MH data increased their confidence in patient diagnoses (round 2: mean 3.8, SD 0.4; and round 3: mean 3.5, SD 0.8), helped them set priorities during visits (round 2: mean 3.5, SD 0.5; and round 3: mean 3.3, SD 1.2), impacted treatment recommendations (round 2: mean 3.5, SD 0.5; and round 3: mean 3.2, SD 1.0), and facilitated collaboration with patients on treatment decisions (round 2: mean 3.2, SD 0.8; and round 3: mean 3.3, SD 0.8). Except in round 2, these providers were unlikely to agree that CAT-MH data helped them assess patients’ response to treatment (round 1: mean 2.8, SD 1.3; and round 3: mean 2.7, SD 1.2).

Regarding questions about equity, mental health care providers consistently reported that the CAT-MH was not benefiting all patients equally (round 1: mean 1.4, SD 0.5; round 2: mean 1.3, SD 0.5; and round 3: mean 1.5, SD 0.5). For TA-related questions—which explored whether CAT-MH data helped these providers better understand their patients, develop a common understanding of treatment goals, and build mutual trust—responses averaged between 1.6 (SD 0.5) and 3.0 (SD 0.6) and generally trended upward over time.

Feasibility scores decreased slightly over the 3 rounds ( round 1: mean 3.6, SD 0.5; round 2: mean 3.5, SD 0.5; and round 3: mean 3.3, SD 0.8). Mean sustainability scores, as measured by the mental health care providers’ sense that benefits would outweigh challenges and that workflows would become more manageable over time, were stably neutral to positive, ranging from 3.2 (SD 0.4) to 3.8 (SD 0.4).

### Data Integration

**Figure 1:**
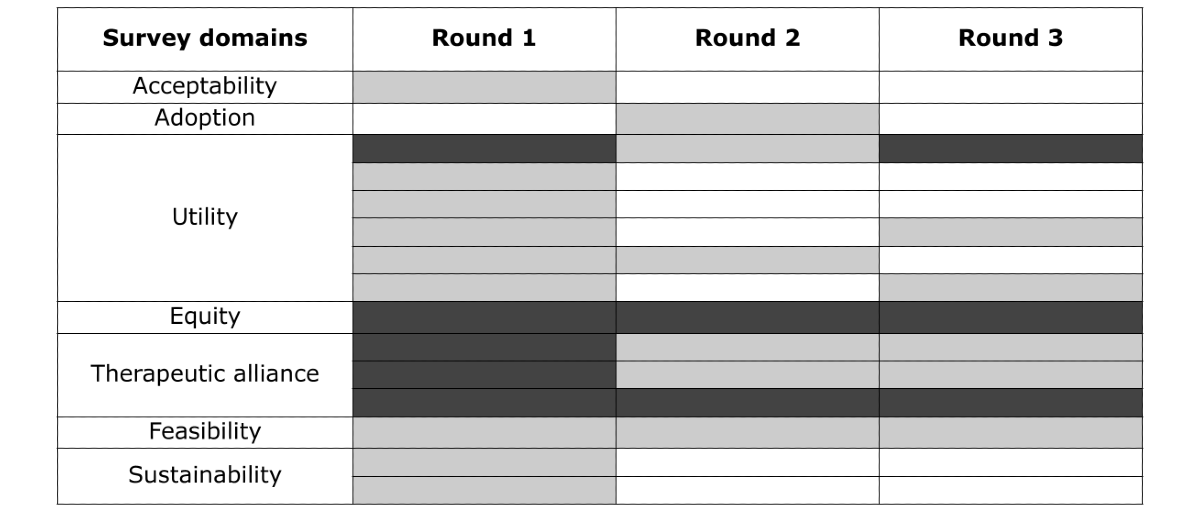
Heat map of quantitative survey results to inform qualitative focus groups. The heat map represents the authors’ qualitative assessment of quantitative survey results, including distributions of answers within each survey round and perceived distance from “ideal implementation” (eg, in the case of adoption). “Good” response profiles were coded in white, “bad” implementation outcome profiles were coded in dark gray, and neutral response profiles were coded in light gray. Each row corresponds to responses for the survey questions listed in Table 2, in order of appearance.

The study leads organized survey findings in a heat map to identify priority areas for the focus group guide (Figure 1). Figure S2 in Multimedia Appendix 1 shows how the study leads examined the distribution of individual item responses over time to flag responses that generally signaled “good” (white), “neutral” (light gray), or “bad” (dark gray) implementation outcomes. “Bad” response profiles, particularly when consistent over time, were prioritized for exploration in focus groups.

Table S1 in Multimedia Appendix 1 provides a high-level description of how the simplified quantitative results were used to select specific CSA subdomains for the focus group guide as well as to generate the questions about equity and TA that emerged from the survey responses. Sample focus group prompts are also presented in Table S1 in Multimedia Appendix 1. Key focus group guide decisions made based on these data included prioritizing equity prompts for each CSA stage, narrowing to CSA subdomain questions with the most relevance to the study context and quantitative findings, and eliciting narratives about the impact of MBC on TA.

Data integration also occurred during qualitative data analysis by incorporating equity as an implementation outcome for valence analysis at each CSA stage (rather than as a stand-alone theme) and coding TA as a stand-alone domain (ie, a level 1 factor). The first decision was made because provider responses indicated unique equity challenges at each CSA stage, and we felt that this was important to disaggregate in valence coding. The second decision was made because the relationship between MBC and TA emerged as more complex and overarching than we felt was captured within the existing CSA stages or implementation outcomes.

### Qualitative Focus Groups

#### Valence Coding Results

Valence coding results are presented by focus group domain. CSA stages of MBC and implementation outcomes (acceptability, appropriateness, feasibility, and equity) are presented in Table 3. The TA domain and its subdomains (MBC impacts TA and TA impacts MBC) are presented in Table 4. Providers gave the most feedback about the *Collect* stage (83 excerpts), followed by *Share* (68 excerpts) and *Act* (28 excerpts).

Within *Collect*, feedback by outcome was mixed, but the overall valence was negative (83 excerpts; mean valence −0.77, SD 0.53), largely driven by very negative ratings for feasibility (mean valence −0.89, SD 0.31) and equity (mean valence −0.96, SD 0.20); ratings for acceptability (mean valence −0.64, SD 0.70) and appropriateness (mean valence −0.39, SD 0.72) were less negative.

*Share* had a relatively neutral valence (68 excerpts; mean valence 0.03, SD 0.91). Provider experiences mostly revealed instances of neutral to positive acceptability (mean valence 0.29, SD 0.90) and appropriateness (mean valence 0.19, SD 0.85) paired with lower feasibility (mean valence −0.33, SD 0.91). Although there were experiences pointing to equity barriers in the *Share* stage (mean valence −1.00, SD 0.00), they were fewer in number than in the *Collect* stage (3/68 excerpts, 4% for *Share* vs 26/83 excerpts, 31% for *Collect*).

**Table 3:** Qualitative valence coding results by Collect-Share-Act (CSA) domain from focus groups and implementation outcome^a^.

CSA domains from focus groups	Acceptability	Appropriateness	Feasibility	Equity	Overall^b^
Collect, mean (SD)	−0.64 (0.70)	−0.39 (0.72)	−0.89 (0.31)	−0.96 (0.20)	−0.77 (0.53)
Collect Excerpts (n=83), n (%)	11 (13)	18 (22)	28 (34)	26 (31)	83 (100)
Share, mean (SD)	0.29 (0.90)	0.19 (0.85)	−0.33 (0.91)	−1.00 (0.00)	0.03 (0.91)
Share Excerpts (n=68), n (%)	21 (31)	26 (38)	18 (26)	3 (4)	68 (100)
Act, mean (SD)	1.00 (0.00)	0.25 (0.89)	−0.07 (1.00)	−1.0 (0.0)	0.11 (0.96)
Act Excerpts (n=28), n (%)	4 (14)	8 (29)	14 (50)	2 (7)	28 (100)

^a^Valences are from −1 to 1, with −1 being a maximally negative valence and 1 being a maximally positive valence.

^b^Overall means and SDs were calculated using all valence data within each qualitative focus group domain (eg, all *Collect* excerpts).

**Table 4:** Qualitative valence coding results by for therapeutic alliance (TA) domain from focus groups^a^.

TA domain valence results	MBC^b^ impacts TA	TA impacts MBC	Overall^c^
Values, mean (SD)	−0.23 (0.87)	0.13 (0.83)	−0.13 (0.86)
Excerpts (n=30), n (%)	22 (73)	8 (27)	30 (100)

^a^Valences are from −1 to 1, with −1 being a maximally negative valence and 1 being a maximally positive valence.

^b^MBC: measurement-based care.

^c^Overall means and SDs were calculated combining both categories of TA domain results (eg, MBC impacts TA and TA impacts MBC)

Providers had neutral to favorable attitudes toward their experiences within the *Act* stage (28 excerpts; mean valence 0.11, SD 0.96), with generally positive acceptability and appropriateness valences (mean 1.00, SD 0.00; and mean 0.25, SD 0.89, respectively). The feasibility valence was neutral to negative (mean −0.07, SD 1.00), while 2 (7%) of the 28 excerpts had a negative equity valence (mean −1.00, SD 0.00).

There were 30 excerpts related to TA (mean valence −0.13, SD 0.86), which revealed instances of MBC impacting TA (n=22, 73%) as well as of TA impacting MBC (n=8, 27%). When mental health care providers shared examples of MBC impacting TA in this safety-net setting, experiences were mixed, but overall resulted in a net negative valence (mean valence −0.23, SD 0.87). Experiences of TA impacting or influencing MBC were also mixed; yet, they resulted in an overall net positive valence (mean valence 0.13, SD 0.83).

#### Drivers of Valence Coding Results by Domain

Table 5 presents representative quotations; and the subsections that follow summarize the factors contributing to positive, negative, or neutral valence within each domain.

**Table 5:** Representative quotations from qualitative analysis of provider focus groups by domain and implementation outcome.

Domains and implementation outcomes			Representative quotations
**Collect**			
	Acceptability	“And the person essentially was like, I’m not filling out any more of those. So that was a drastic response.” [Participant 2] “He sent a message saying that he would not be filling this out because he had too many experiences of filling questionnaires out, and they’re not used, and it’s a waste of his time.” [Participant 4]	
	Appropriateness	“Probably because of the acuity of the patients that I’m meeting with, it doesn’t feel as relevant of a conversation...[later]...it’s not a top priority compared to the other things that I’m trying to accomplish, which is mostly stabilization.” [Participant 2]	
	Feasibility	“Patients don’t know what it is. They, they get a lot of surveys, especially from primary care. Um. And yeah, so that’s, there’s a, a lot of that education that happens when, when a patient brings it up or if, if I bring [Computerized Adaptive Test for Mental Health] up.” [Participant 5]	
	Equity	“I’ve had some folks from non [...] US western cultural backgrounds who have gotten some strange scores on the perceived—the, um, how are they calling the psychosis module?...And I do think some of it is just culturally they, their answers are very normative, um, within their upbringing and, and not within kind of the, I think some of the bias in the, in the questions.” [Participant 5] “I have to agree with [Participant 3] that it feels like the goals are a little skewed. Um, you know, uh, helpful for us helping a health care system treat a whole population, um, may be important, but it’s hard to figure out how you, how that translates individually patient to patient.” [Participant 6]	
**Share**			
	Acceptability	“I had not had on my radar any symptoms of psychosis for this person. And the measurement...came up as high risk or severe something. So I was able to ask more specific questions about that and actually determined that there was a level of paranoia and was more attuned to potential psychotic symptoms. If I had not seen the CAT-MH, I wouldn’t have gone down that road of assessing that with them, and then a couple months later, the person actually expressed suicidal ideation and was hospitalized. So it was helpful to have the measurements to talk through more of the symptoms and also because there was kind of a lack of affect around what the person was reporting. The CAT-MH helped me recognize the possible severity of the symptoms.” [Participant 2] “I’ve also had a couple strong experiences that stick out in my mind of what I perceive as like, harm that has occurred, um, from taking the test. And those have mostly been in patients with PTSD and I’m, I’m curious if [Participant 7] and [Participant 5] have had those experiences too. But, um, one who was very distraught because her PTSD score was not high enough and so it invalidated her experience.” [Participant 1]	
	Appropriateness	“Um, and the, the test results of the CAT-MH, as far as I can tell, like, they, they’re somewhat black-and-white as far as like what goes into the ADHD diagnostic category, um, versus, you know, attentional issues that are anxiety based or whatever. And I, and that I think maybe could reinforce like a more black-and-white understanding of where this could come from for the patient.” [Participant 3]	
	Feasibility	“I’m gonna put a plug in for, uh, as a psychiatrist, I...there’s not enough time in your visits. I’m just putting it out there. I mean there’s patients who report it, psychiatrists report it, there’s just not enough time.” [Participant 7]	
	Equity	“I think patients who may have more therapy experience...who have more experience thinking about their emotional experiences, like, are probably more likely to um, be able to talk about it.” [Participant 2]	
**Act**			
	Acceptability	“There have been a couple of instances where people’s scores have improved and it’s been like an opportunity for, kind of, some celebration, which I think is nice. And it’s nice to have this sort of like, this objective feel, you know, to, to their improvement, um, in a way that I think that’s probably been the most meaningful. I, um, you know, that it’s been for, for my treatment relationships.” [Participant 3] “I would say it’s generally not changed treatment plans.” [Participant 5] “I feel we’re pretty far still [from realizing the full potential benefits of measurement-based care], speaking from my experience and my team’s experience.” [Participant 6]	
	Appropriateness	“He was in remission for both [conditions] and then finally we, his, he did his CAT-MH and it, you know, the ADHD piece came up and it was something we had talked about when I first started working with him, but it was just so, the last priority, and, and that was a nice reminder of um, you know...it, it led to some good conversations and some book recommendations and it’s, it’s been a topic that we’ve, we’ve been kind of delving into.” [Participant 5]	
	Feasibility	“We started a medication. Maybe like three months later, whatever it was, those scores lessened and we’re like, okay, let’s continue this medication. And it felt very, like, simple and clean.” [Participant 1]	
	Equity	“The patient population that uses the tool more, um, and, and therefore that like has a little bit more economic stability. Um, computer literacy. Um, generally more often White, more often middle class, more often identified, more often with their diagnosis and their, their mental health care. Um, those are the folks who I see as, like, uh, uh, benefiting more and also putting in the work, you know, to, to engage with it more.” [Participant 3]	
**TA^a^**			
	MBC^b^ impacts TA	“I thought it was interesting because sort of like, um, as a...a possible kind of point of contention between us...I didn’t want to introduce, like, a stimulant for instance.” [Participant 3] “I think there’s been this sort of pivot away from relationship-based care...in community psychiatry that’s been this sort of, like, painful decades-long thing...and I think that I have sort of seen these...quantitative, computer-based assessment triage tools as like a piece of that shift.” [Participant 3]	
	TA impacts MBC	“I have felt very much like I had to side on, like I and I do side on their side, but I felt like I had to join with them to kind of, like, protect them against this CAT-MH thing that happened to them. Um, and try to separate myself from that test.” [Participant 1] “One of my highest risk people I think ever...scored just so beautifully low on everything...We’re getting the wrong score on the CAT-MH and I had a conversation with her and she said, ‘Oh, it’s easy to figure these out...I don’t wanna get hospitalized.’...We had a good conversation about it...she’s chronically suicidal, so she’s always gonna score really high on those measures and she’s like, hospitalization doesn’t help.” [Participant 5]	

^a^TA: therapeutic alliance.

^b^MBC: measurement-based care.

#### Factors Contributing to Positive, Negative, or Neutral Valence Within Each Domain

##### Collect

###### Acceptability

Excerpts with positive acceptability valence often referenced mental health care providers’ positive buy-in for engaging in MBC. Examples of excerpts with negative acceptability valence included mental health care provider reports of patient difficulty completing the measure or strong negative reactions to the measure, providers feeling that nothing they said to patients improved patient engagement in measure completion, providers questioning the validity of the measure, and providers’ low MBC buy-in.

###### Appropriateness

Examples of excerpts with positive appropriateness valence included mental health care providers feeling that administration frequency was suitable. Examples of excerpts with negative appropriateness valence included concerns about MBC’s fit across the system (eg, mental health care providers feeling that encouraging patients to engage in MBC was not a good use of their limited time with patients with complex needs, especially for those in crisis).

###### Feasibility

Examples of excerpts with negative feasibility valence included mental health care providers reporting patients with higher symptom burden having trouble with the length of the MBC measure. They also include concerns that the centralized MBC approach (ie, a multidiagnostic MBC symptom tool selected by clinical leadership, administered centrally via the patient portal, and at the same frequency for all patients as determined by the leadership) created confusion for patients and providers because patients simultaneously received other types of electronic surveys from the health system.

###### Equity

The mental health care providers in this study reported a number of observed barriers to the equitable implementation of MBC data collection for their patient populations, including varying health literacy and English language literacy; lack of availability in languages other than English and Spanish; exclusion of patients not using the electronic patient portal; and measure completion challenges for patients working multiple jobs, experiencing life stressors, living with greater functional impairment, or less actively engaged in their mental health treatment. These providers shared salient examples of the CAT-MH, and MBC in general, not being a good fit for patients from non-Western cultural backgrounds; for example, one mental health care provider shared that a patient became very upset after receiving scores indicating high psychosis-related symptom burden during a period of acute grief after the death of multiple family members. The provider felt that the score may have been influenced by factors related to how this patient experienced grief and loss in their culture, rather than being reflective of psychopathology; however, the patient was lost to follow-up, and the provider wondered whether it was because of the MBC results. Some mental health care providers theorized that MBC might help improve equity at the population health level but were not sure how equity “translate[d] individually patient to patient.”

##### Share

###### Acceptability

Examples of excerpts with positive acceptability valence included instances in which discussing results together helped the mental health care provider identify a new clinical concern. One provider described being prompted by unexpectedly high CAT-MH scores to “ask more specific questions,” subsequently realizing that the patient may have psychosis symptoms. The combination of the high severity scores and the patient’s “lack of affect around what [they were] reporting” gave the provider the clues needed to explore a new diagnosis. Examples of excerpts with negative acceptability valence often included instances of scores not matching a patient’s subjective experience. Some mental health care providers expressed concerns that patients were harmed when their MBC results showed lower severity scores than their subjective experience, which they perceived as invalidating. Mixed acceptability (neutral valence) excerpts were also common.

###### Appropriateness

Examples of excerpts with positive appropriateness included that some mental health care providers felt that patients’ ability to view results via the patient portal before their visit was a good fit with their practice, allowing patients to formulate questions before the discussion about the scores. The providers in this study had mixed reports (neutral valence) about numeric scores, which they felt, for example, could sometimes be interpreted as too definitively diagnostic or pathologizing. They found it challenging to find the right moment to introduce a discussion of the results and felt conflicted about whether using the visit time to do so was valuable, given other urgent priorities.

###### Feasibility

Some mental health care providers with shorter appointments (ie, psychiatrists and psychiatric nurse practitioners) liked having only summary CAT-MH scores available because it increased the feasibility of reviewing data during visits. Other mental health care providers (mostly therapists) felt that not having individual item responses made it harder for them to interpret the scores with their patients. The providers in this study reported numerous examples where interpreting MBC data together was feasible, particularly within an established care relationship. Negative or mixed drivers of feasibility included factors complicating communication about the measure and results, including that (1) patients routinely received other electronic survey requests and were sometimes confused about which “survey” their mental health care providers were referencing, (2) mental health care providers did not always know whether patients had viewed their scores, (3) providers sometimes forgot to bring up new scores during visits even when they were available, and (4) providers and patients sometimes disagreed on the relevance of the results or the amount of time that should be allotted to discussing them during the visit.

###### Equity

Mental health care providers reported that it was harder to make time during visits to discuss CAT-MH results for patients experiencing more life stressors, medical complexity, or challenges arriving to appointments on time.

##### Act

###### Acceptability

Mental health care providers expressed mixed acceptability in the *Act* domain, and all reported that the implementation effort seemed far from realizing the full potential benefits of MBC. In the rare instances where providers did report using MBC data to help make treatment decisions, their experiences generally revealed positive acceptability; for example, a provider described the act of appraising patient progress via repeated measure administration as creating an opportunity to jointly celebrate patient progress that may otherwise have been missed. Most providers in the study reported that their treatment plans did not change based on the MBC results.

###### Appropriateness

Appraising the data together to contextualize and prioritize treatment goals was seen as an appropriate application of MBC. One mental health care provider described how a patient felt validated when the CAT-MH flagged their longtime concerns about possible attention-deficit/hyperactivity disorder and how the patient and provider were able to discuss the results and agree to focus first on more urgent clinical needs and revisit the symptoms of inattention once these were under better control.

###### Feasibility

The most positive feasibility examples in the *Act* domain included instances in which mental health care providers could use the CAT-MH results to make decisions around adjusting medications, for example, when the CAT-MH showed symptom improvement after starting a medication and facilitated a decision to continue the selected medication. Others reported more mixed experiences (neutral scores), for example, when the results did not align with patient reports, which made it more challenging to decide whether any adjustments to treatment were needed.

###### Equity

Mental health care providers who reported concerns about equity in the *Act* domain shared that while they could see the potential benefit of MBC for equity at the system level (eg, by guiding the investment of scarce resources into programs that served identified patient needs), they did not see evidence that MBC was reducing health inequities for their individual patients. In large part, this was due to the fact that engaging with MBC was a prerequisite to benefiting from it, and the people most likely to engage with the CAT-MH seemed to be those from more privileged backgrounds and thus in less need of care optimization.

##### TA Analysis

###### MBC Impacting TA

Our analysis indicated a bidirectional relationship between MBC and TA that was both positive and negative. MBC positively influenced TA when MBC strengthened the bond between patient and clinician (eg, by highlighting when to celebrate patient progress) and when MBC helped patients and their mental health care providers jointly prioritize certain mental health concerns over others in the short and long term (eg, agreeing to prioritize other mental health conditions before possible attention-deficit/hyperactivity disorder). On the flip side, MBC negatively influenced TA when mental health care providers saw quantitative assessments as a “pivot” away from relationship-based care; when it was difficult to reach consensus between provider and patient about the accuracy, meaning, or implications of the MBC results; and when patients had very strong, negative reactions to the process of measure completion or the results themselves, with their frustration sometimes directed toward providers. Some mental health care providers reported having to “talk down” patients and highlighted instances in which they had to make significant efforts to preserve or repair TA after a rupture occurred due to invalidating results. Some clinicians also noted that taking the time to encourage CAT-MH completion or discuss the results came at the cost of time for other therapeutic and relational aspects of care.

###### TA Impacting MBC

Mental health care providers shared examples in which strong TA helped mitigate potential negative patient experiences with MBC, although whether this translated into increased or decreased uptake of MBC varied by case. Where mental health care providers had strong TA with patients, they reported being able to navigate the *Share* and *Act* stages, even when the results seemed inaccurate; for example, a provider for a patient considered high risk recognized that the measure scores were inappropriately low and discussed with the patient their reasons for underreporting symptoms (namely the patient’s wish to avoid hospitalization). In this case, strong TA helped the clinician overcome MBC’s limitations.

#### Participant Suggestions for Adaptations to MBC Implementation (No Valence Applied)

Textbox 1 presents mental health care providers’ suggestions for improvement (excerpts without valence). When mental health care providers in our focus groups suggested MBC adaptations that were entirely speculative, they received appropriate level codes and an “N/A” valence. In the *Collect* stage, mental health care providers suggested that patients might complete the CAT-MH at higher rates if they could choose a subset of questions in their assigned modules (this suggestion was not implementable within the current workflow). Relevant to *Collect* and TA, mental health care providers also suggested that MBC might build trust more effectively if introduced specifically when diagnostic clarity was needed, rather than routine symptom progress monitoring only. Mental health care providers hypothesized that the *Share* stage of MBC might be more acceptable if scores were not shared with the patient until the visit time, allowing providers to facilitate interpretation and discussion. They also suggested that ensuring equity at the *Share* stage might require standardizing when the results are discussed in patient encounters (eg, at the start of every visit). Most providers in the study stated that they were not changing existing treatment plans in response to MBC data, in accordance CSA’s *Act* stage. Mental health care providers felt that they would feel more confident deciding when to change treatment plans based on MBC data if they were given with decision support tools, such as shared decision-making scripts incorporating the MBC results; concrete options for changing treatment plans within existing system and patient constraints; data about how their treatment decisions impacted patient outcomes; and MBC target goals for specific populations where it was likely to have the biggest impact.

**Participants’ suggestions for improvement (excerpts without valence).**

- “I would love to know, like, is my prescrib[ing]...am I making the right decisions? And I’m sure that analytics could be used in some, you know, in some way to inform me about that.” [Participant 3]
- “I, I, you know, I wonder if it would build trust if it was something I introduced at a moment where there was a need for diagnostic clarification...There are times where patients are asking me and they’re wanting to know like, what do you think? And could this be this? Or, you know, we’re trying to clarify the diagnosis together. And I’ll say...It could help us if you filled that out and then we could talk about it afterwards.” [Participant 6]
- “Instead of having the whole range of different diagnoses, what if we picked one of the ones that we have—you know—our patients who are suffering with the most, they’re, they’re...that we’re not seeming to really affect much change. And what if we...honed in on just one piece and then, you know, would that make it easier for us to use...easier for the, those patients who need it to use, and for us to see different results?” [Participant 6]
- “Um, so I think if we screen for it, we need to have tools and things to help them when they screen positive for those things...And so I’m feeling, this is separate from CAT-MH, but also I guess related to measurement-based care in some ways maybe. So wanting to have the resources in place if we’re gonna ask the questions so it’s not further traumatizing.” [Participant 1]

## Discussion

### Principal Findings

MBC literature has highlighted its potential and observed benefits, but there has been little documentation of real-world MBC implementations in underresourced health systems serving patients from diverse backgrounds and clinical profiles. This pragmatic mixed methods study adds to the literature by inviting feedback from a group of mental health care providers with >1 year of MBC experience who work with a heterogeneous group of patients with high needs in a safety-net setting.

By beginning with sequential surveys over 6 months and ending with focus groups that were deeply informed by the patterns that emerged from the surveys, the mixed methods used in this study allowed the data collection and interpretation to yield a gestalt understanding of MBC experiences of mental health care providers that could not have been obtained from quantitative or qualitative approaches alone. While the quantitative surveys were helpful in understanding the ongoing barriers and high-level perceptions of mental health care providers in the system, they could not answer why some markers of implementation outcomes did not improve—and in some cases worsened—as providers gained experience with MBC. Similarly, the strength and persistence of the converging survey responses pertaining to health equity influenced our decision to ask about health equity concerns at each CSA stage, which in turn influenced our ability to present a richer view of health equity in MBC than may have been possible in prior literature. Using the qualitative focus groups to explore the findings about TA from the quantitative data helped us uncover a richer understanding of the bidirectional relationship between TA and MBC, which we had not seen discussed in prior literature.

One key finding from our study was that the CSA model, which was originally developed with individual patient-provider dyads in mind, has limitations when applied at the health system level. Unlike individual practitioners, health systems tend to rely on centralized, coordinated processes to implement MBC. In our setting, department leadership decided on a single measure that would be sent to all patients, and administrative support staff sent the measure via the patient portal at a set time before upcoming visits. While these decisions were intended to streamline MBC data collection and hence improve feasibility for mental health care providers, they ultimately precluded some of the original CSA model’s recommended *Collect* substeps from being performed; for example, providers could not discuss why they chose the MBC measure for the specific patient or involve the patient in selecting measures relevant to their treatment plan, both of which are identified in the CSA model as ideal practices. Instead, they were limited to introducing MBC as an innovative approach to care and explaining why the specific measure (ie, the CAT-MH) had been selected by the organization.

Our study also highlights the ways in which a centralized approach to MBC implementation may have mixed impacts on its acceptability. First, some of the mental health care providers in our focus groups identified moral injury associated with collecting data that quantified their patients’ distress when they felt that they did not have clear guidance on how to adjust treatment in response, especially given their patients’ very real resource constraints.

Second, to comply with the 21st Century Cures Act [42], our organization’s MBC workflow shared results directly and instantaneously with patients via the patient portal. While prior studies indicate that patients generally prefer immediate release of most test results [43], our focus groups revealed that some patients had strong negative reactions to the MBC results they had viewed on their own—reactions that their mental health care providers then had to mitigate during appointment times. One possible solution to this problem would be to administer MBC measures immediately before a session, with the patient in the office waiting room. This approach would comply with the 21st Century Cures Act while also giving clinicians the opportunity to guide the initial conversation about the measure results.

Finally, because our system chose a single MBC measure that drew from an extremely large question bank, individual item responses were not available in the electronic health record. While implementation leaders chose this design to facilitate IT feasibility [44], clinicians’ inability to review which items their patients endorsed likely impacted the ways in which they could be expected to engage with and respond to the CAT-MH results. Future studies should assess whether mental health care providers in similar settings who are presented with data from simpler MBC measures have more success with discussing and acting on the data in collaboration with their patients.

Drawing from these observations, our study indicates that researchers seeking to use the CSA model to guide MBC implementation in mental health systems may need to add components to the model that capture where clinic- or organizational-level factors can better support patients and care providers at each stage. Our work suggests that efforts to centralize the process in the service of feasibility may unintentionally undermine MBC acceptability for patients and mental health care providers alike. Anticipating where centralization may add friction and mitigating these effects are likely necessary to facilitate successful MBC implementation in contexts such as ours.

Another key finding from our study was that MBC may exacerbate health disparities, which contrasts with the foundational premise that MBC has the potential to enhance mental health equity [4]. Our study participants observed that MBC was being implemented unevenly across patient subgroups (as reflected in the survey data), which seemed to be driven largely by barriers to the *Collect* stage (as reflected in the focus group data). The stated drivers of unequal implementation echo those observed in patient-reported outcomes literature, namely language barriers; functional impairment driven by disease burden; differences in health and technology literacy and access; and differences by patient age, race and ethnicity, or gender [23,45,46]. In other words, because completing the CAT-MH was a necessary step before the data could be used to adjust treatment, the patients who could not or would not complete it—who tended to be marginalized across ≥1 domains—were unable to benefit from the treatment approach.

These observations are critical, given the value that implementation leaders in diverse safety-net settings place on offering equitable, culturally relevant mental health care [47-49]. Our findings, which align with prior research that emphasizes the importance of developing measures and treatment approaches that are culturally appropriate [21,50], suggest that a direct focus on practical implementation and equity may be needed in future MBC research. However, acting on these findings may be increasingly difficult as health systems that are subject to value-based payments face pressure to develop centralized MBC processes that limit patient-mental health care provider dyads from finding the most appropriate measure or choosing to abstain when the selected measure is not a good fit. These areas warrant further investigation.

Finally, while prior evidence suggests that MBC can enhance TA, our findings suggest that the relationship between MBC and TA is more complex and not wholly positive. Strong baseline TA seemed to enhance mental health care providers’ ability to proceed through the CSA stages, including collecting and assessing the accuracy of the data, working with patients to resolve potential discrepancies between the data and the patient’s narrative report, making sense of the data, and deciding how to proceed. However, when MBC was perceived as a threat to the TA, the clinicians in our study prioritized preserving or repairing their relationship with the patient over collecting MBC data and applying the results to treatment. In addition, mental health care providers were more enthusiastic about MBC when the data confirmed their preexisting diagnostic formulations. Conversely, the providers we spoke to tended to trust their clinical judgment over MBC data when the two were discordant. Furthermore, when the data differed from the patient’s own explanatory model for their illness, mental health care providers perceived MBC as burdensome, sometimes leading to short- or long-term complications for TA. Bearing this complex relationship in mind, future implementation efforts may benefit from directly addressing the tension MBC can cause for TA, and future implementation research can assist these efforts by studying specific strategies to address this tension. Therapeutic ruptures may strengthen TA in the long run [51], but time-constrained safety-net mental health care providers would benefit from training on how to prepare for the ruptures that MBC may cause as well as institutional support to ensure time and opportunity for repair.

### Strengths, Limitations, and Future Directions

This small-scale hypothesis-generating study had many strengths. First, we identified challenges and potential adaptations at each CSA stage in a diverse safety-net setting. Second, we intentionally included equity as a level 2 valence code, a critical implementation outcome that is often undercharacterized in implementation research. Third, we recruited mental health care providers with at least 1 year of real-world safety-net MBC experience and studied their responses longitudinally via sequential mixed methods to intentionally prioritize “informational richness” [39]. Specifically, we used purposeful sampling of “information-rich” cases [39], selecting mental health care providers from a health system that had implemented MBC 1 year prior and devoted significant resources to ongoing training and quality improvement activities in an effort to improve MBC uptake. Therefore, all participants in the study had experienced the same training and implementation activities as a group over this time period (emphasis on sampling for highly overlapping context, experience, and role in each case). In this sense, informational richness was prioritized a priori [39] with both the purposive sampling method and the use of the repeated surveys before the focus groups. In other words, the repeated quantitative data were used to hone the focus group questions over time to ensure maximum relevance for the study participants.

Although our focus group sample size was small (n=7), it was sufficient for phenomenological studies of lived experience, where 3 to 6 participants are generally considered adequate [39]. As the mental health care providers in our study not only participated in MBC trainings and gained experience with MBC through their 1-on-1 patient care but also engaged in ongoing team meetings and grand rounds related to MBC over this time period, any provider who participated in the study was aware of not just their own experience of MBC implementation but also that of their colleagues. This type of broad understanding of the phenomena emerged during focus groups, where open dialogue was encouraged. By balancing pragmatic data collection that minimized participant burden with a focus on information richness from mental health care providers who had a great deal of experience with the specific phenomena in an urban safety-net mental health clinic, this study adds significant value to directions for future research.

This study also had several limitations. Although we did not intend to test hypotheses, the relatively small study sample may not have been representative of the full group of mental health care providers in our department. Providers who participated were more likely to be female (5/6, 83%) compared to those who did not participate (13/19, 68%). In addition, among participants, 50% (3/6) were clinical psychologists or psychiatrists or psychiatric nurse practitioners and 50% (3/6) were social workers, compared to 42% (8/19) and 58% (11/19), respectively, among those who did not participate. While we did not gather feedback about reasons for nonparticipation from each eligible care providers, clinician burnout was noted to be prevalent at the time of the study. Given this context, it is possible that the clinicians who did not participate had lower morale, which could mean that the feedback we gathered was more positive than it would have been with broader representation. Of note, our response rate of 27% (7/26) is in line with prior research about specialist participation in web-based surveys [52]. Nevertheless, scaling up similar study designs would likely require efforts to reduce barriers to clinician participation even further. With greater levels of participation, future studies could assess whether the care settings or patient populations with whom different mental health care providers work correlated in any way to their response to MBC implementation.

Among the clinicians who did participate, there was considerably more feedback on the *Collect* stage than the *Share* or *Act* stages of MBC. This was likely because relatively few patients completed >1 CAT-MH. To the extent that we were able to gather feedback on the *Share* stage, some of the participants in our study identified the fact that individual item responses were not visible in the electronic health record as a limitation in the implementation design, although for others this was noted to increase feasibility.

Another potential limitation is that the study leads, AMP and LCR, worked in the same organization as the participants. In theory, this may have suppressed people’s willingness to participate in the study or share candid feedback about their experience with the measure. However, these effects were likely mitigated by the fact that the researchers interacting with the potential and enrolled participants had no supervisory relationship with them. Moreover, the recruitment and informed consent processes were designed to highlight the steps being taken to ensure that participant feedback remained anonymous to anyone in leadership or with a supervisory relationship with participants.

Finally, the study context may have impacted findings in a way that is important to consider. The health system where this study was conducted attracts mental health care providers who have a psychodynamic orientation, which may not lend itself as easily to MBC as other more structured treatment modalities. Health care providers in our system are also drawn to working with diverse patients as well as those considered disadvantaged, who may require additional coaching to overcome cultural and educational barriers to filling out written screeners as part of care. Furthermore, some of the challenges we identified may have been specific to the chosen MBC measure and implementation design: a computer-adaptive tool that was sent out through a centralized, system-wide messaging platform and administered remotely on patients’ own electronic devices. As such, the directions for future research that we identified may be more relevant for care settings and implementation designs that share some of these characteristics.

### Conclusions

Our study found that implementing MBC in safety-net health systems poses significant challenges that warrant further study and likely need to be addressed to ensure that all patients benefit equally from this evidence-based treatment approach. Our qualitative analysis highlighted unanticipated challenges at each CSA stage, for which health systems have limited guidance at this time. These were particularly evident in the *Collect* stage, although the mental health care providers in our study had less experience with the *Share* and *Act* stages, limiting the depth of feedback in these areas.

The rich data from this mixed methods analysis also highlighted the negative as well as positive ways in which MBC can impact TA. Our results point to a need for more research and clearer applied strategies about how to prioritize TA amid MBC implementation so that uptake is enhanced, and unintended psychological harm to patients is avoided.

Finally, our findings revealed bidirectional concerns regarding equity, namely that MBC may be inequitably implemented in safety-net settings and may also exacerbate rather than reduce health disparities among patient subgroups.

These findings, which are meant to uncover important areas for further attention and study, are timely in the current policy environment. Interest in psychiatric MBC is growing, and reimbursement is increasingly being tied to collecting and demonstrating improvements in patient-reported outcomes. Future research should explore which tools and how much customizability will improve MBC adoption in safety-net systems as well as which components of each CSA stage can be centralized to improve MBC feasibility without compromising equity or TA. Moving forward, it will be essential to address existing knowledge gaps about how to implement MBC equitably to avoid disproportionately impacting both the financial and ethical imperatives of safety-net health systems.

## Appendix

Multimedia Appendix 1 Supplementary Figure S1 (Explanatory Sequential Study Design of Provider Experiences of MBC), Supplementary Figure S2 (Example of Individual Item Analysis Used to Generate Heatmap of Quantitative Survey Results), Supplementary Table S1 (Applying Patterns from Quantitative Findings to Narrow and Refine Focus Group Guide Approach), and Supplementary Table S2 (Qualitative Coding Tree Used for Qualitative Coding of Focus Group Data).

## Abbreviations

CAT-MH: Computerized Adaptive Test for Mental Health
CSA: Collect-Share-Act
MBC: measurement-based care
TA: therapeutic alliance

## Data Availability

The focus group guide is available from the corresponding author on reasonable request. The datasets generated or analyzed during this study are not publicly available, in accordance with the participant research protections at our institution. Limited, deidentified data summaries or tools may be available from the corresponding author on reasonable request and subject to institutional policies on research protections.
