# Supplementary material for: Mental Health Care Provider Experiences of Remote Measurement-Based Care Rollout in an Urban Safety-Net Psychiatry Department: Three-Site Mixed Methods Hypothesis-Generating Implementation Study
Source: JMIR Form Res. 2025 Sep 5;9:e71570. doi: 10.2196/71570 (PMC12449665; doi:10.2196/71570)
Supplement: Multimedia Appendix 1 [file formative_v9i1e71570_app1.docx]

**SUPPLEMENTARY MATERIALS**

**Supplementary Figure S1.**

*Explanatory Sequential Study Design of Provider Experiences of MBC*

**
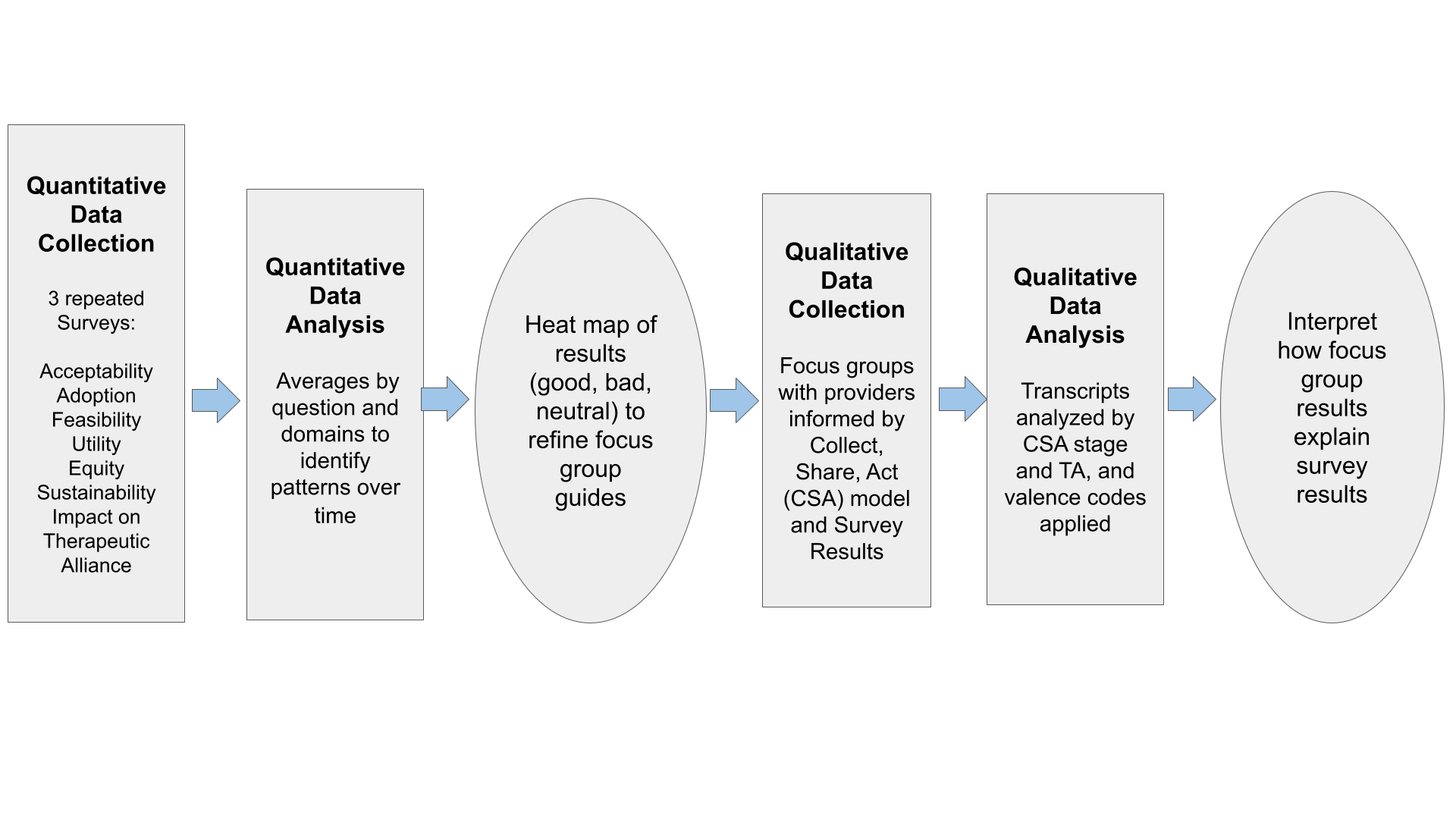
**

**Notes:** MBC = Measurement-Based Care; CSA = Collect, Share, Act Model. Mixed Methods figure adapted from: Creswell, A Concise Introduction to Mixed Methods Research (2015). See reference list.

**Supplementary Figure S2.**

*Example of Individual Item Analysis Used to Generate Heatmap of Quantitative Survey Results*

**
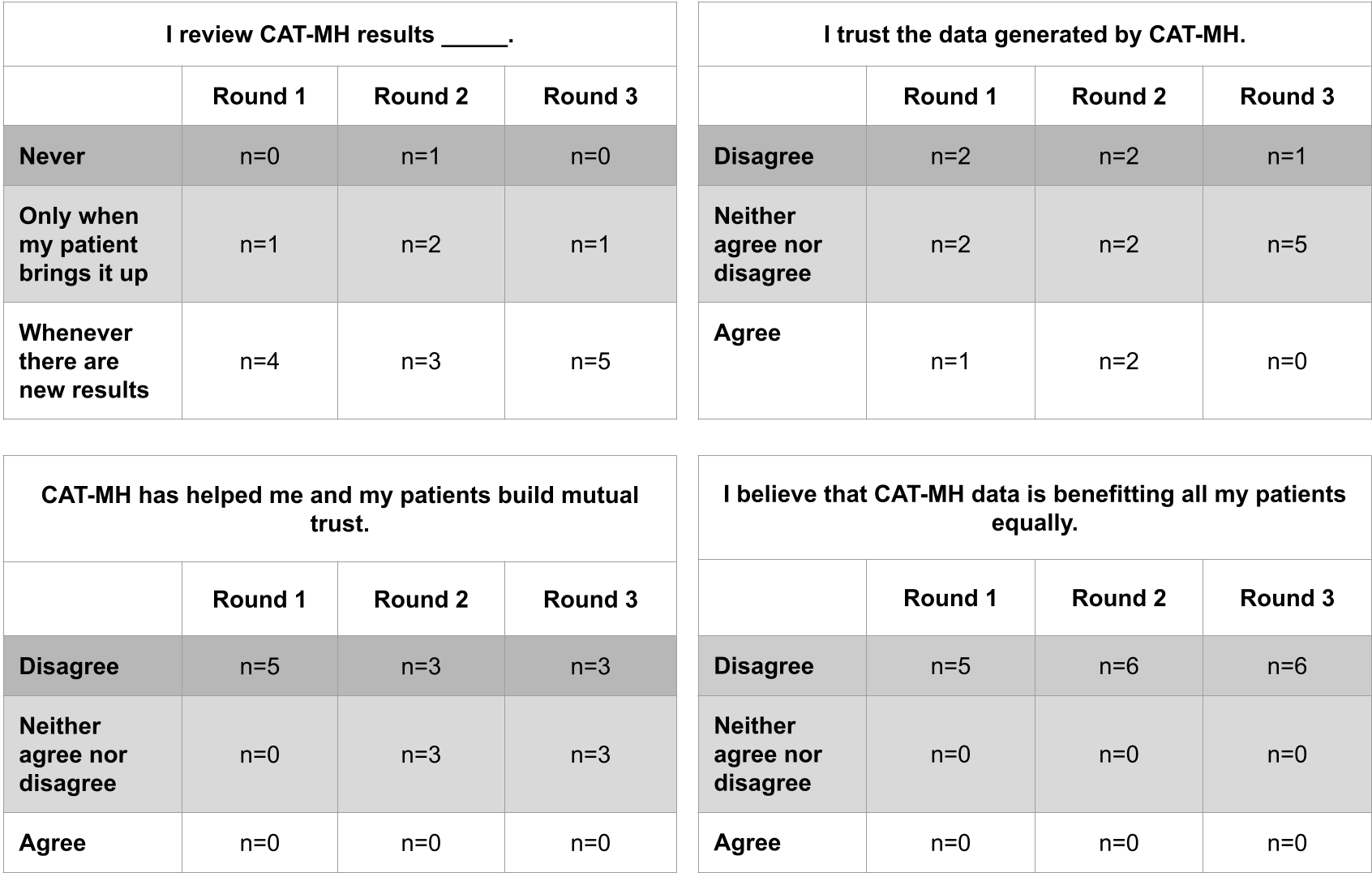
**

**Supplementary Table S1.**

*Applying Patterns from Quantitative Findings to Narrow and Refine Focus Group Guide Approach*

| **Phase** | **Simplified Observations from Surveys (Takeaways)** | **Decisions for Focus Group Guide** | **Example Focus Group Prompts** |
| --- | --- | --- | --- |
| Collect | Provider acceptability of CAT-MH was neutral, and their trust in the data accuracy did not increase.  Providers did not think MBC was benefitting all patients equally; this result was unanimous and consistent. | Focus questions on process sub-steps related to:   - (*CSA Sub-Domain)* Explaining rationale for MBC - (*Equity Question*) Offering equal opportunity for patients to engage in MBC data collection | “How do you see your role in the process of introducing MBC to patients?”  “What do you think still needs to happen to ensure all patients can take the CAT-MH?” |
| Share | Providers' sense that discussing CAT-MH in visit was feasible decreased slightly. Meanwhile, they were neutral to negative regarding whether using CAT-MH improved common understanding and mutual trust with patients.  Providers did not think MBC was benefitting all patients equally; this result was unanimous and consistent. | Focus questions on process sub-steps related to:   - (*CSA Sub-Domain)* Verifying that the score reflects the clients’ subjective sense of mood, symptoms, and/or functioning - (*Equity Question*) Offering equal opportunity for patients to view and discuss MBC measures with providers - (*Therapeutic Alliance*) Understanding how the MBC “Share” phase impacts Therapeutic Alliance | “Overall, how has your experience been with reviewing data together with patients?”  “How has it impacted your work when CAT-MH scores have confirmed or been contrary to a working diagnosis?”  “What do you think still needs to happen so that all patients have equal opportunity to engage in meaningful discussion with providers about their results?”  “How has CAT-MH impacted your relationship with patients?” |
| Act | Providers’ sense that using CAT-MH helped set priorities for visit or impact treatment recommendations or collaborative decision making improved slightly over time, but remained relatively neutral.  Providers did not think MBC was benefitting all patients equally; this result was unanimous and consistent. | Focus questions on process sub-steps related to:   - (*CSA Sub-Domains)* Appraising meaning of data in terms of patient’s trajectory; determining if adjustments should be made collaboratively - (*Equity Question*) Offering equal opportunity for patients to benefit from the MBC processes - (*Therapeutic Alliance*) Understanding how engaging in shared decisions around treatment using MBC impacts Therapeutic Alliance | “Have you used CAT-MH data with patients to modify treatment?”  “Can you share some examples where you have used CAT-MH data to engage in collaborative decision-making with your patients?”  “What do you think still needs to happen so that all patients have an equal chance in having their treatment being improved based on the scores?” |

**Supplementary Table S2.**

*Qualitative Coding Tree Used for Qualitative Coding of Focus Group Data*

| **Level 1 Codes**  (Qualitative Focus Group Domain) | **Level 2 Codes**  (Outcomes) | **Level 3 Codes**  (Direction of Influence on Outcome) | **Level 4 Codes**  (Subdomains of Level 1 Code) |
| --- | --- | --- | --- |
| Collect  Share  Act | **Acceptability:**  *Providers like how [C, S, A ] is working*  **Appropriateness:** *Providers think we should be doing*  *[C, S, A] as indicated*  **Feasibility:**  *Providers are able to do [C, S, A]*  **Equity:**  *Providers find [C,S,A] implementation equitable; or impact of [C,S,A] is equitable* | Valence Rating:  Examples or factors that increase (+1), decrease (-1),  or have mixed impact (0)  on Level 2 outcomes within each CSA step  N/A codes applied to speculative examples. | **Collect Stage Subdomains**  Explain: Introduce MBC to patient  Select: Discuss why you chose measures; involve the client when possible  Administer: Measure regularly and repeatedly as standard care  Centralized Workflow or System-Level Collect Activities*  **Share Stage Subdomains**  Report: Report data to client, explain what it means, highlight relevant areas  Verify: Verify score reflects clients’ sense of mood, symptoms, or functioning  Capture: Capture data in medical record/progress note  Centralized Workflow or System-Level Share Activities*  **Act Stage Subdomains**  Appraise: Assess meaning of the data with respect to trajectory  Determine: Decide whether adjustments should be made  Brainstorm: Engage collaboratively with client to discuss possible options  Choose: Collaboratively agree on plan of action  Timeframe: Agree on timeframe for re-evaluating  Document: Document discussion and decisions in client chart  Centralized Workflow or System-Level Act Activities* |
| Therapeutic Alliance | MBC Impacts Therapeutic Alliance  Therapeutic Alliance Impacts MBC* | Valence Rating:  Direction of relationship between MBC and TA, or TA and MBC  (-1, 0, +1, n/a) | MBC comes between provider and patient to threaten or create rupture in TA  MBC enhances TA between patient and provider  TA enhances the ability to perform one of the steps of the CSA*  TA enhances positive impact, or mitigates unintended consequences, of MBC*  TA itself, or concerns about preserving TA, negatively impacts MBC processes in CSA steps* |

Notes: *Indicates an inductive code derived from the data; not a-priori (deductive). Subdomains of Collect, Share, Act stages are drawn from the CSA model itself (Barber & Resnick, 2022); we shorten the full description of these clinical process sub-steps for brevity of presentation.

**References:**

Creswell J. W. (2015). A concise introduction to mixed methods research. Sage.

Barber J, Resnick SG. Collect, Share, Act: A transtheoretical clinical model for doing measurement-based care in mental health treatment. Psychological services. 2023;20(S2):150.
